# Deconstructing Insight: EEG Correlates of Insightful Problem Solving

**DOI:** 10.1371/journal.pone.0001459

**Published:** 2008-01-23

**Authors:** Simone Sandkühler, Joydeep Bhattacharya

**Affiliations:** 1 Commission for Scientific Visualization, Austrian Academy of Sciences, Vienna, Austria; 2 Department of Neurophysiology, Center for Brain Research, Medical University of Vienna, Vienna, Austria; 3 Department of Psychology, Goldsmiths, University of London, London, United Kingdom; Claremont Graduate University, United States of America

## Abstract

**Background:**

Cognitive insight phenomenon lies at the core of numerous discoveries. Behavioral research indicates four salient features of insightful problem solving: (i) mental impasse, followed by (ii) restructuring of the problem representation, which leads to (iii) a deeper understanding of the problem, and finally culminates in (iv) an “Aha!” feeling of suddenness and obviousness of the solution. However, until now no efforts have been made to investigate the neural mechanisms of these constituent features of insight in a unified framework.

**Methodology/Principal Findings:**

In an electroencephalographic study using verbal remote associate problems, we identified neural correlates of these four features of insightful problem solving. Hints were provided for unsolved problems or after mental impasse. Subjective ratings of the restructuring process and the feeling of suddenness were obtained on trial-by-trial basis. A negative correlation was found between these two ratings indicating that sudden insightful solutions, where restructuring is a key feature, involve automatic, subconscious recombination of information. Electroencephalogram signals were analyzed in the space×time×frequency domain with a nonparametric cluster randomization test. First, we found strong gamma band responses at parieto-occipital regions which we interpreted as (i) an adjustment of selective attention (leading to a mental impasse or to a correct solution depending on the gamma band power level) and (ii) encoding and retrieval processes for the emergence of spontaneous new solutions. Secondly, we observed an increased upper alpha band response in right temporal regions (suggesting active suppression of weakly activated solution relevant information) for initially unsuccessful trials that after hint presentation led to a correct solution. Finally, for trials with high restructuring, decreased alpha power (suggesting greater cortical excitation) was observed in right prefrontal area.

**Conclusions/Significance:**

Our results provide a first account of cognitive insight by dissociating its constituent components and potential neural correlates.

## Introduction

Having a cognitive problem may be defined as the gap between where we are now (initial state) and where we want to be (goal state, solution), yet we do not know how to find a way to cross this gap [1]. Often a problem is solved by proceeding along a continuous smooth solution path. For instance, algebraic problems usually involve a distinct but fixed number of sequential steps between initial state and goal state which form a smooth solution path. On the other hand, if there is a discontinuity in thinking or solution path [2], an insight or transformative thinking is needed, which primarily involves a mental restructuring that often leads to a subjective “Aha!”-an experience in which the problem solver becomes suddenly and unpredictably aware of the solution. There exist numerous anecdotes of such moments of insight, such as Archimedes' discovery of the law of buoyancy, Newton's finding of the law of gravitation or Kekulé's discovery of the ring structure of benzene [3]. Our intuitive understanding of this insight phenomenon or insightful problem solving seems to be quite distinct, but psychologists have not yet agreed upon a single definition of insight [4], [5], [6], [7], [8], [9], [10]. However, there are general agreements that insightful problem solving can be characterized by four salient features as follows:

1. Mental impasse: There is a high probability that the problem solver experiences an impasse in the process of solving the problem, wherein the solver is mentally stuck on an unsuitable construct of the problem and fails to progress further [11]. The mental impasse may be a force directing problem solving efforts and providing resistance to finding new interpretations of the problem that must be overcome or it may merely be a temporal blockage of information retrieval needed for reaching the goal [4], [12]. A number of researchers have reasoned that an excessive focus on inappropriate or irrelevant cues hinder the problem solver to recognize the possibly obvious solution [13], [14], [15], [16], [17]. The solution space constrained by the problem solver can also be prejudiced if a word or an object is used only in its most habitual usage and association [5], [8]. Due to this set effect and/or exhausting the available options and resources, the problem solver is likely to experience a mental impasse since she is unable to make further progress and subsequently she gives up [18]. From an information processing point of view, the system has reached a limiting point at mental impasse: any new possible options or interpretations from long-term memory are blocked from further processing within working memory. Attentional processes usually serve as a “general gatekeeper” for manipulating information within working memory by exerting top-down control over which information is the most relevant and may occupy the limited space [19]; thus, mental impasse may be caused by an “attentional overload”.
2. Restructuring: This is a mechanism by which the problem solver breaks out of mental impasse [20]. It is a transition from an initial inappropriate and thus misleading representation of a problem and state of not knowing how to proceed in solving a problem to a state of knowing how to solve it [8], [9], [10], [21]. Restructuring is made possible either by internal retrieval processes which search long-term memory for concepts which can be utilized to reinterpret the available knowledge in the problem space [22] or by the availability of external cues [15], [23]. There is, however, an ongoing debate on the underling mechanisms of restructuring involved in insight problem solving, with different researchers proposing two oppositional processes [24]. According to the first hypothesis, restructuring is a controlled, conscious and attention-intense process [22], [25], and the second hypothesis suggests an automatic and subconscious recombination where relevant pieces of information in long-term memory are automatically and subconsciously recombined [24], [26], [27], [28], [29]. This leaves the problem solver unable to report what enabled him to overcome mental impasse and successfully restructure the problem representation [30], [31].
3. Deeper understanding: An insight is a form of deeper or more appropriate understanding of the problem and its solution [4], [6], [7], [9], [32]. Citing the Oxford dictionary, insight is “the capacity to gain an accurate and intuitive understanding” [33]-so a true insight must lead to a correct solution [29], [34].
4. Suddenness: An insight is often perceived by the problem solver as being spontaneous or sudden and without any predictable forewarning [5], [6], [8], [10], [35], which is the often reported subjective “Aha!” experience.

Up-to-now research on insightful problem solving has largely been confined within the domain of behavioral psychology [4], [5], [6], [7], [8], [9], [10], [11], [12], [21], [30], [32], [35], [36], while there are only a few attempts to identify the underlying neural mechanisms [37], [38], [39], [40]. However, only the post-solution [37], [38], [41], [42] or pre-problem presentation [40] periods were investigated, rather than the actual process of problem solving, i.e. the pre-solution period. Thus from these studies no conclusion can be drawn about the neural processes underlying the solution pathway. One study [39] investigating the pre-solution period classified insight if the problem solver reported an “Aha!” experience with solution. But the other key features of insight such as restructuring and mental impasse were not addressed. The simple fact that a problem solver experiences an “Aha!” tells us little about the cognitive processes which brought about solution (and that experience). That is, if one does not obtain a fine-grained behavior pattern or thought progression as the problem solver solved the problem, then one can offer rather limited information about the cognitive and neural mechanisms underlying insight.

So, there is a paucity of knowledge about the neural correlates of various features of cognitive insight phenomena analyzed in a unified framework. Therefore, the main aims and objectives of this study were to investigate the following questions related to the four salient features characterizing insightful problem solving: (1) Mental impasse: Can we find potential neural correlates, that reflect an “attentional overload”, which blocks further processing of new information from long-term memory, and the effort to search for available options and resources prior to mental impasse? Further, can any neural precursor to a successful break of functional fixedness on an unsuitable problem representation be detected? (2) Restructuring: What is a potential neural correlate of restructuring? Can we identify the brain responses which would support a controlled, conscious and attention-demanding process or rather an automatic, unconscious recombination mechanism? (3) Deeper understanding: Are there any differences in the EEG signals immediately prior to a correct versus a false positive “more appropriate” understanding of the solution? (4) Suddenness: Can the EEG results from Jung-Beeman and others [39] be confirmed, who reported gamma activity at right temporal region beginning at 300 ms before the insightful solution? Are there any further characteristic neural signatures of the problem solver's feeling of suddenness and obviousness? (5) Finally, can we reveal any relationship between the process of restructuring and “Aha!”?

As stimuli, we used the compound remote associate problems [43], where each problem consists of three test words (e.g., back, clip, wall) and the subject needs to generate a solution word (paper), which forms a valid compound word or phrase with each of the three test words (paperback, paperclip, wallpaper). These problems have been frequently used in studies on insight and creativity [39], [40], [43], [44], [45]. Though they are much simpler than the classical insight problems, they do share the key properties of these problems, thus calling for the involvement of similar cognitive processes in solving more complex and information-rich insight problems [45]. Further, there are also some specific advantages of using the compound remote associate problems since they (i) can be solved rapidly thus enabling the experimenter to record multi-trial data from a single session, (ii) can be solved without any domain-specific knowledge or expertise, and (iii) exist in a wide variety yet are structurally homogeneous. Finally, one can easily create hints, that again are all of the same structure and that may inform the subject at a meta-level that a reinterpretation of the problem is needed [15]. Behavioral data was obtained by registering ratings of the constituent features on a trial-by-trial basis.

The most relevant neural activities underlying complex cognitive processes including problem solving occur at the level of tens to hundreds of milliseconds [46], [47], [48]. Modern imaging studies, such as functional magnetic resonance imaging (fMRI), are found to be very useful to localize brain functions, but at the cost of low temporal resolution, which is in the range of a few seconds [49]. Since insight and its constituent features are dynamic and short-lived in time, EEG seems perfectly suitable to address these questions due to its excellent millisecond range temporal resolution and still offers reasonable spatial resolution for signals in the cerebral cortex. Further, neuronal oscillations are aptly represented by the EEG signals [50] and it is widely documented that large scale brain oscillations in various frequency bands is modulated by a diverse range of tasks in human cognition (see [51], [52], [53] for reviews). Thus, in order to address our objectives in a common framework and with systematic fashion, we conducted a multivariate EEG experiment in which human participants repeatedly solved verbal problems that could create a small scale experience of insight. EEG signals were extensively analyzed in the space×time×frequency domain with a multivariate nonparametric cluster randomization test.

## Results

### Behavioral performance

Table 1 summarizes the behavioral performance of the participants solving the compound remote associate tasks. It lists the median number (with range) of trials per participant for each of the twelve possible outcomes (see Materials & Methods). Table 2 shows the percentages of correct and incorrect trials with and without hint. The participants correctly solved 38.8% (mean, SD = 9.6) of all compound remote associate task trials without a hint and gave an incorrect solution to 11.2% (SD = 8.0). After hint presentation, 39.4% (SD = 8.1) of all solutions were correct and 4.7% (SD = 6.1) of the hints led to an incorrect solution. 55.9% (SD = 11.9) of all post-hint trials could not be solved within the provided time limit.

**Table 1:** Median (range) number of trials per subject that were in each of the conditions.

	Median (range) number of trials
**correct solution** (without hint)	38 (24–54)
**false positive** or incorrect **solution** (without hint)	7 (2–32)
**mental impasse**	31 (0–57)
**timeout** (of the initial 45 s)	12 (0–55)
**correct solution after mental impasse** (post-hint)	13 (0–31)
**timeout after mental impasse** (post-hint)	16 (0–39)
**correct solution after initial timeout** (post-hint)	2 (0–20)
**post-hint timeout** (after initial 45 s timeout)	6 (0–34)
**correct solution with no restructuring**	15 (0–31)
**correct solution with full restructuring**	4 (0–21)
**non-sudden correct solution**	1 (0–8)
**sudden correct solution**	17 (4–32)

**Table 2:** Percentage of correct and incorrect trials.

	Percent of trials	
	without hint	with hint
**Correct solution**	38.8 (9.6)	39.4 (8.1)
**Incorrect solution**	11.2 (8.0)	4.7 (6.1)

Mean (SD) percentage of compound remote associate problems for which the subjects gave correct or incorrect (false positive) solutions without and with hint.

The participants solved 21 out of 22 (median, range: 18–22) control tasks, where the correct solution was presented, correctly and missed responding within the time-limit in 0 (0–2) cases. Further, the participants solved 10 out of 11 (5–11) control tasks, where an incorrect solution was presented, correctly and missed responding within the time-limit in 0 (0–5) cases.

### Correlation between Rating of Suddenness and Rating of Restructuring

**Figure 1:**
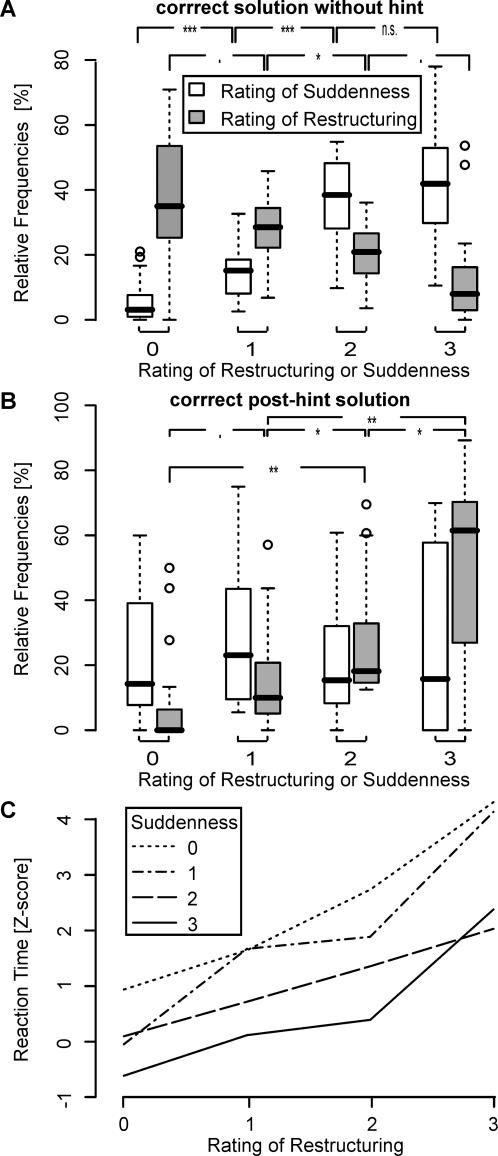
Relationship between restructuring of the problem and suddenness of the solution. (A) Relative frequencies as a function of subjective rating of restructuring and suddenness for correct solutions without hint. The higher the rating of suddenness, the more frequent it was. For restructuring ratings the relative frequency distribution was reverse. The higher the rating of restructuring, the less frequent it was. (B) As in (A), but for correct post-hint solutions. After hint presentation the distributions of relative frequencies changed. The higher the rating of restructuring, the higher were the relative frequencies. The four levels of suddenness rating were equally often chosen. (C) Interaction Plot of reaction time with ratings of suddenness and restructuring. Mean reaction times, expressed in Z-scores on the ordinate, are plotted as a function of subjective rating of restructuring for 4 different levels of suddenness. Dotted line: gradually approached correct solution (rating of suddenness = 0); dash-dotted line: rating = 1; dashed line: rating = 2; solid line: solution appeared abruptly without any conscious forewarning (rating = 3). The interaction plot displays, that the longer the required time for a correct solution was, (i) the higher was the subjective rating of restructuring and (ii) the lower was the suddenness feeling. Thus, the shortest reaction times were found for sudden solutions with no restructuring and the longest reaction times were found for non-sudden solutions with full restructuring. ’.’, ’_*_’, ’_* *_’ and ’_* * *_’ correspond to significance levels *p*<0.1, *p*<0.05, *p*<0.01 and *p*<0.001 of a Wilcoxon signed rank test, respectively.

For correct solutions without hint the relative frequencies of the four levels of rating of suddenness were significantly different (Friedman rank sum test, *p*<0.0001): the higher the rating of suddenness, the more incidences we observed (Fig. 1*A*). The least frequent was a suddenness rating of 0-we only observed it in 3.1% (0–21.1) of all trials, irrespective of the rating of restructuring. For the rating of restructuring we found an opposite significant effect (Friedman rank sum test, *p*<0.001): the higher the restructuring rating for correct solutions without hint, the fewer incidences we monitored (Fig. 1*A*). For example, 23.2% (mean, SD = 14.6) of all correct solutions were sudden and involved no restructuring and 0.0% (0–41.5) of all correct solutions were sudden and involved full restructuring. For 55.6% (SD = 18.2) of the correct solutions without hint participants reported a high feeling of suddenness (≥2) and at the same time low involvement of restructuring (≤1), at least at the consciousness level. For 23.3% (SD = 17.1) of the correct solutions problem solvers gave a simultaneous high rating of restructuring (≥ 2) and suddenness (≥2).

The mean Spearman rank correlation coefficient *ρ* between the ratings of restructuring and suddenness was significantly negative (*ρ* = −0.39, one-sided t-test, *t*_18_ = −5.17, *p*<0.0001). This negative correlation effect was significantly attenuated (two-sided paired t-test, *t*_18_ = −7.21, *p*<0.0001) for correct post-hint solutions (*ρ* = −0.08, *t*_18_ = −0.98, *p* = 0.34). After hint presentation, which should help to restructure the problem representation successfully, the relative frequencies of the four different restructuring levels were again significantly different (Friedman rank sum test, *p*<0.0002; Fig. 1*B*). However, after hint presentation, we observed higher frequencies for greater subjective restructuring ratings. The relative frequencies of the suddenness rating were more uniformly distributed (Friedman rank sum test, *p* = 0.78; Fig. 1*B*).

### Dependency of restructuring and suddenness rating on reaction time

Next, we analyzed the interaction effect between the rating of suddenness, the rating of restructuring and the reaction time for correct solutions without hint. The results based on the linear mixed effects model (see Materials & Methods) indicated that the reaction time differed for different ratings of restructuring (*F*_3,641_ = 120.1, *p*<0.0001) and suddenness (*F*_3,49_ = 24.3, *p*<0.0001). Strong linear and weaker quadratic effects of restructuring (*p*<0.0001 and *p*<0.08 respectively) and suddenness (*p*<0.0001 and *p*<0.08) were found. Particularly, we observed (see Fig. 1*C*): the longer the required solution time for a correct solution was, (i) the higher was the subjective restructuring rating and (ii) the lower was the suddenness feeling. The median reaction time for a correct solution with specific ratings of suddenness and restructuring can be seen in Table S2. There was no significant restructuring×suddenness interaction effect on the reaction time (*p* = 0.4). For correct post-hint solutions we observed attenuated linear effects of restructuring (*p*<0.1) and no linear effects for suddenness (*p* = 0.37) on reaction time.

### Mental impasse

32.0% (SD = 18.3) of all compound remote associate tasks led to a mental impasse and the mean time spent to solve the problem before registering mental impasse was 27.9 s (SD = 5.1).

### EEG data

We compared four pairs of conditions in the electrode×time×frequency space as follows. For each comparison, we performed paired (two-sided) t-tests, thresholded them at *p* = 0.01 and searched for electrode×time×frequency clusters. Finally we assessed the significance between conditions with a nonparametric Monte Carlo randomization cluster test correcting for the multiple comparisons between electrodes and time-windows (see Methods for details).

### Mental impasse

One of the reasons behind a mental impasse is functional fixedness or mental set as proposed by Gestalt psychologists. For compound remote associate problems, this is equivalent to the situation when a subject was fixated on generating obvious semantic associations with test words which led to the application of inappropriate information and to the retrieval of incorrect target words. Insight may occur after this period of frustration in which the subject did not make any progress. But how does the period of mental impasse differ from similar periods of unsuccessful searching for the solution?

**Figure 2:**
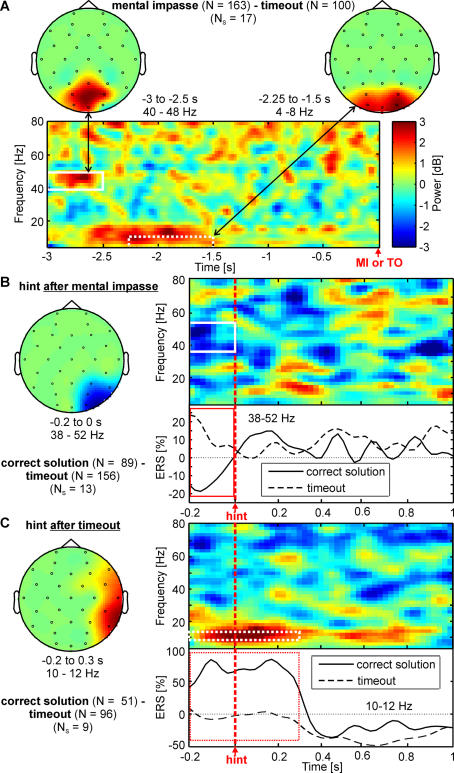
Significant clusters of the nonparametric cluster randomization tests for mental impasse and timeout. (A) Comparison of mental impasse with timeout trials. Top left: topographical map of the parieto-occipital gamma band cluster displaying significant differences between the baseline-corrected spectral power of mental impasse and timeout with *P*<0.005. Top right: topography of the occipital theta band cluster for the same comparison. Bottom: Pseudo-color coded time-frequency representation in decibel (dB) of the difference between mental impasse and timeout averaged over electrodes PO3, Oz and PO4. Red color indicates higher spectral power for mental impasse and blue color indicates higher spectral power for timeout. Mental impasse response or timeout is at t = 0 s. (B) Comparison of the immediate pre- and post- hint presentation time period after reaching a mental impasse between hints that led to a correct solution versus those that led to a timeout trials. Topographical map on the left shows the right parieto-occipital gamma band cluster, displaying significant (*P*<0.04) differences. Top right: Pseudo-color coded, grand-averaged time-frequency representation of all electrodes that comprise the significant cluster. Bottom: grand-averaged gamma frequency event-related synchronization (ERS) averaged for both conditions between 38 and 52 Hz and for the same electrodes as in the time-frequency representation. The red rectangle marks the zone of significance (*p*<0.01). Red color indicates higher spectral power for correct solution trials and blue color indicates higher spectral power for timeout trials. (C) As in (B) but comparing the immediate pre- and post- hint presentation time period of hints after timeout that led to a correct solution versus those that led to a further timeout. The dotted red rectangle marks the zone of significance with *p*<0.05. The white rectangles with solid and dotted lines in the grand averaged time-frequency representation plots show the time-frequency zones of significance with *p*<0.01 and *p*<0.05, respectively.

Therefore, first we compared mental impasse (*N* = 163) with initial timeout (*N* = 100), and found two clusters at parieto-occipital and occipital electrodes. One earlier strong gamma band effect (mental impasse>timeout) from −3 to −2.5 s (40–48 Hz; *P*<0.005, *N_S_* = 9) and a later theta frequency band cluster (4–8 Hz; *p*<0.05, *P*<0.04) from −2.25 to −1.5 s before mental impasse (Fig. 2*A*). Secondly, we contrasted the initial processing of hints after mental impasse that led to a successful utilization and thus correct post-hint solution (*N* = 89) with those that led to an unsuccessful utilization and later timeout (*N* = 156, Fig. 2*B*). From −0.2 to 0 s before hint presentation timeout trials showed ERS and correct solutions displayed ERD (38–52 Hz; *P*<0.04, *N_S_* = 13) in right parieto-occipital brain regions (PO4, P8, P4, O2).

On the other hand repeating the same comparison, but for the immediate pre- and post hint presentation period of hints that were presented after timeout (Fig. 2*C*), yielded a significant cluster in the upper alpha frequency band (10–12 Hz). More precisely, we found for subsequent correct solutions after timeout (*N* = 51) as compared to a further timeout (*N* = 96) a strong upper alpha ERS in right temporal regions (correct solution>timeout; *p*<0.05; *P*<0.04, *N_S_* = 9) from −0.2 to 0.3 s after onset of hint presentation.

### Restructuring of the Problem

**Figure 3:**
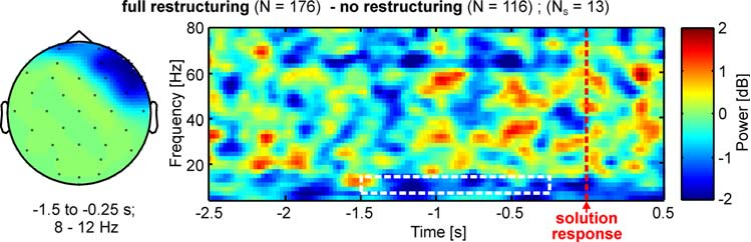
Significant cluster of the nonparametric cluster randomization test for correct solutions involving full restructuring versus those involving no restructuring of problem. Left: topographical map of the right prefrontal alpha (8–10 Hz) frequency band cluster displaying significant (*P*<0.075) differences between the baseline-corrected spectral power of correct solutions with full restructuring versus those with no restructuring. Right: Grand averaged time-frequency representation of the difference between correct solutions with full restructuring and those with no restructuring over all electrodes that comprise the significant cluster. Red color indicates higher spectral power for correct solutions with full restructuring and blue color indicates higher spectral power for correct solutions with no restructuring. Time of solution response is at 0 s marked by dotted red line. The white dotted rectangle marks the time-frequency zone of significance (*p*<0.05).

Solving a problem with insight often requires restructuring of the problem representation with a focus on the new cognitive structure leading to the final correct solution. Restructuring is also necessary for overcoming mental impasse. To investigate the underlying neural correlate of restructuring, we compared correct solutions without hint that were post-hoc rated as having involved full restructuring (*N* = 176) with those that were rated as having involved no restructuring (*N* = 116). We found a right pre-frontal effect from −1.5 to −0.25 s before solution in the alpha frequency band (8–12 Hz; no restructuring>full restructuring; *p*<0.05; *P*<0.075, *N_S_* = 13; Fig. 3).

### Deeper understanding

**Figure 4:**
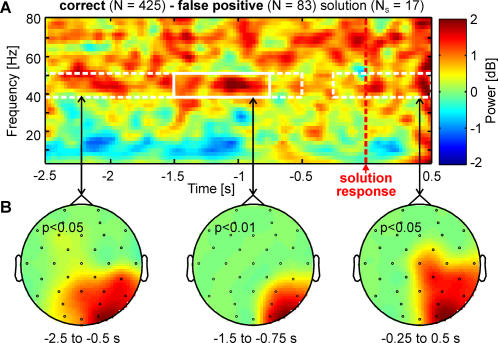
As Figure 2*A* but comparing correct identifications of solutions versus incorrect (false positive) ones. (A) Grand averaged time-frequency representation of the difference between correct and false positive solutions over electrodes O2, PO4, P4 and P8. (B) Topographies of the three significant clusters at right parieto-occipital electrodes due to the gamma frequency band (40 − 50 Hz) effect. Left: P<0.002. Middle: *P*<0.005. Right: *P*<0.02. Red color indicates higher spectral power for correct solutions and blue color indicates higher spectral power for false positive solutions. Time of solution response is at 0 s marked by red dotted vertical line.

Insightful problem solving involves a form of deeper or more appropriate understanding of the problem and its solution. Thus, an insightful solution must be a correct solution. Here, we compared correct (*N* = 425) with incorrect or false positive solutions (*N* = 83) without hint to investigate if there are any differences between the neural processes leading to a correct solution (and thus a true deeper understanding of the solution) and an incorrect solution (and thus a false positive understanding of the problem and its solution). We observed the main difference at right parieto-occipital area in gamma band (correct>false positive; 40–50 Hz; *P*<0.005, *N_S_* = 17; Fig. 4) from −1.5 to −0.75 s. Increasing the pre-specified threshold to *p* = 0.05 revealed two further right parieto-occipital gamma band clusters (40–50 Hz, correct>false positive), one earlier cluster from −2.5 to −0.75 s (*P*<0.002) and one later effect from −0.25 to 0.5 s after solution response (*P*<0.02).

### Suddenness of the Solution

Insightful solutions often occur unpredictably and abruptly thus leading to the notion that insight is the product of a sudden process. After verbalizing the solution at the end of each trial, the participants indicated whether the solution emerged suddenly inside the mind like a sudden illumination of a light-bulb (rating of suddenness = 3 on a 4-point scale) or the solution gradually appeared with conscious control like dimming up a light bulb (rating of suddenness = 0; see Methods).

**Figure 5:**
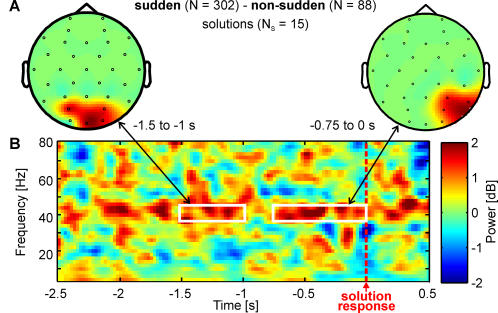
As Figure 2*A* but comparing sudden versus non-sudden solutions. (A) Left: topography of the bilateral parieto-occipital gamma frequency band cluster (38–44 Hz, P<0.003) displaying significant differences between the baseline-corrected spectral power of sudden and non-sudden solutions. Right: topographies of the right parieto-occipital gamma band cluster (38–44 Hz, P<0.04) displaying significant differences between the baseline-corrected spectral power of sudden and non-sudden solutions at two different times. (B) Grand averaged time-frequency representation of the difference between sudden and non-sudden solutions for the strongest representant PO4 (in terms of significance), common to both clusters. Red color indicates higher spectral power for sudden solutions and blue color indicates higher spectral power for non-sudden solutions. Time of solution response is at 0 s marked by red dotted vertical line.

We compared sudden (*N* = 302) with non-sudden solutions (*N* = 88) and found two significant gamma frequency band clusters (38–44 Hz) in the parieto-occipital area. The earlier effect (from −1.5 to −1 s before the solution) was more localized at occipital regions (PO3, Oz, O2 and PO4; *P*<0.003, *N_S_* = 15, sudden>non-sudden; Fig. 5*A*, left topography plot) and the later (from −0.75 to 0 s) cluster was primarily at right parieto-occipital region (*P*<0.002, sudden>non-sudden; Fig. 5*A*, right topography plot). The right hemisphere parietal cluster resembles very much (in frequency and location) the effect that we observed for the correct versus false positive solution comparison (Fig. 4*B*).

## Discussion

A crucial limitation of earlier research on insight using insight problems is the explicit assumption that these problems can only be solved by an insight. Categorizing a problem as an insight problem (or noninsight problem) offers no concrete information about the cognitive processes involved in solving these problems. For example, some of these insight problems could be solved in non-insightful ways [45] yet still with the “Aha!” [54]. So, the association between problem-type and requirement of an insight is much less rigid than usually assumed. Therefore, instead of dwelling on the insightful nature of the problem, we have focused here on the insightful nature of the solution and on obtaining a fine-grained behavior or thoughts pattern by receiving the immediate post-solution ratings of suddenness, restructuring and confidence on trial-by-trial basis.

### Mental Impasse

Several researchers argue that mental impasse is a necessary condition for insight [8], [28], [29] and that the time period of struggling with the problem without achieving a solution is as important as the sudden realization (but also see [2] for a different view). In the compound remote associate task the problem solver is not always able to retrieve all known words from long term memory within the allotted time period. It is reasonable to assume that after a few attempts of retrieval followed by unsuccessful match, the problem solver believes that all originally available and relevant information has already been considered and utilized in the best possible way [29]. This may then lead to a mental impasse.

Our results indicate a neural correlate of mental impasse in parieto-occipital brain regions in the gamma frequency band (Fig. 2*A*). This may suggest that selective attentional processes are accountable for an excessive focus on an inappropriate problem representation, since (i) the primary candidate brain region which modulates selective attentional demand is the posterior parietal lobe [55], [56], and (ii) the primary oscillatory activity which gets modulated by selective attention is the gamma frequency range [57], [58], [59]. Usually the attentional load is externally manipulated by the experimenter, e.g. by increasing the number of moving targets to be attended by the subject [60]. In contrast, in the present paradigm the external stimuli remained the same but the internal attentional demand changed by an increasing number of unsuccessful solution path attempts that needed to be stored in working memory, so that they were not repeated by the problem solver. Since working memory is limited, attention may serve as a “gatekeeper” for this limited space in working memory [19]. Thus the increasing (top-down) control by attention could potentially cause mental impasse due to an “overload”. At right parieto-occipital regions the gamma power was stronger just before hint presentation for mental impasse leading to timeout than for mental impasse leading to a correct solution (Fig. 2*B*). This possibly reflects excessive selective attention which hampers finding the solution even after the hint presentation.

Additionally, we found a theta frequency band effect (Fig. 2*A*), where mental impasse elicited stronger theta frequency band activity (4–8 Hz) over parieto-occipital regions from −2.25 to −1.5 s before moment of mental impasse. Oscillations in the theta band are relevant for diverse cognitive tasks, typically involving some aspects of working memory [61], [62], [63], [64], encoding of new information into episodic memory [65], [66], [67] or navigation [68], [69], [70]. Kahana and others [68] demonstrated in a virtual maze navigation task that theta oscillations were more apparent in more complex mazes and during retrieval phase. Subsequently, it was hypothesized that theta oscillations facilitate encoding and retrieval in memory during a virtual maze learning task [69]. Thus, the observed theta effect is possibly associated with an increased search in the memory space for possible solution words prior to mental impasse.

As can be seen in Figure 2*B,C*, we found significant right hemisphere effects at and immediately before hint presentation that predicted successful hint utilization, irrespective of if the problem solver experienced a mental impasse or if unsuccessful problem solving attempts led to a timeout. These results are consistent with previous studies that have demonstrated the critical role of the right hemisphere in overcoming fixation and finding non-obvious, non-dominant interpretations of words and information in insight problems, that at first glance seem merely remotely relevant [27], [71], [72]. Specifically, Fiore and Schooler [71] showed that hints to insight problems are more effective when presented to the left visual field (i.e. right hemisphere) than when presented to the right visual field (i.e. left hemisphere).

Furthermore, we investigated the preparatory and immediate post hint presentation phase of initial timeout trials (not involving a mental impasse) and found corroborating evidence to an interesting behavioral visual hemifield study by Beeman and Bowden [72]. By adopting a similar compound remote associate task paradigm but with solution related priming, they found that the initial processing pertaining to the solution is active in both hemispheres but fades faster in the left hemisphere because of a fine semantic focus on a misleading interpretation of one of the test words, yet remains persistently active in the right hemisphere due to a broad semantic solution-relevant activation. However, this right hemispheric activation is not likely to reach the level of awareness because it is weak, diffuse and possibly suppressed by stronger, yet misleading, processing in the left hemisphere.

However, what is the neuronal correlate of this sustained suppression of the solution-related right hemisphere activation? Can we find any difference in the neuronal processes between solving attempts that successfully utilized the hint and those that could not? Interestingly, we found for timeout trials that would lead to a correct solution after hint presentation, a strong alpha ERS (10–12 Hz; Fig. 2*C*). Increase in alpha activity is usually associated with a relaxed, less active brain, because alpha power was largest in states with eyes closed, i.e. states without focused attention [73], [74]. There is also evidence that alpha oscillations reflect an active suppression mechanism of non-task relevant cortical brain activity for internally driven mental processes [53], [75], [76], [77]. Jung-Beeman and others [39] observed increased alpha band ( *f_c_* = 9.8 Hz) activity over right posterior parietal cortex prior to insightful solutions and associated it with unconscious solution related processing. The strong alpha ERS observed over right temporal area just before hint presentation indicates an inhibition of right temporal area, which has been associated with the integration of distantly related semantic or lexical information [39]. However, this inhibition is only needed when competing neuronal processes exist. We suggest that the alpha ERS may be related to a weak, unconscious processing of the solution in the right temporal area, consistent with previous research showing that a solution to a verbal problem can be weakly activated in the right hemisphere [27], [44], [78]. With the aid of the hint the initially weakly activated solution related information became intensified and reached the level of awareness. The alpha ERS effect was not found in timeout trials that failed to produce any solution; since no solution-relevant information was active at the first place, nothing had to be suppressed, and so the hint could not help to find the final solution.

### Deeper Understanding

The gamma frequency band activity at parieto-occipital regions was stronger for correct (40–50 Hz) than for false positive solutions (Fig. 4). Interestingly functional fixedness or mental impasse was also associated with increased gamma frequency band activity (40–48 Hz) at right parieto-occipital regions (Fig. 2*A*). A hint presentation was less successful during stronger gamma frequency band activity (38–52 Hz) in parieto-occipital brain areas (Fig. 2*B*). This may indicate that attentional processes (i.e. selective attention) are important to produce and identify a correct solution, but on the other hand excessive amount of selective attention could overload the information processing capabilities and cause impaired performance. Thus, this could explain (i) why the gamma band power of a mental impasse is still higher in comparison to timeout, which possibly reflects the problem solvers excessive focus on an inappropriate problem representation and thus “attentional overload”, which blocks further processing of selecting target words from verbal memory, leading to mental impasse, and (ii) why excessive attention during mental impasse prevents that an external hint can successfully be utilized by the problem solver. One could thus speculate that the degree of gamma band oscillations must remain at an optimal (i.e. sub-maximal) level to maximize the performance.

### Restructuring of the Problem

In the present study the neural correlate of conscious restructuring (full vs. no) was mainly found in the alpha band (8–12 Hz) and in right prefrontal brain regions (Fig. 3). This result is in line with earlier studies. In a large patient study, Miller and Tippett [79] demonstrated that patients with a focal right prefrontal cortex lesion performed poorly while solving problems requiring some sort of restructuring. The prefrontal cortex is also crucially involved in planning in open-ended tasks [80]. Further, in a single-case study, Goel and Grafman [81] demonstrated that a right prefrontal cortex lesion severely impairs the capability to generate solutions for ill-constrained problems and also the capability to restructure or break the mental fixedness. Recent fMRI studies of healthy participants [82], [83] also implicated the right prefrontal cortex in mental restructuring that leads to an abrupt gain of explicit knowledge producing an insight. Altogether the present results strengthen the critical role of right prefrontal cortex in conscious restructuring.

### Suddenness of the Solution

A number of studies [35], [45], [84] assumed Aha!, the feeling of sudden emergence of the solution, as a hallmark of an insight. The present results indicate the neuronal correlate of suddenness (as shown in Fig. 5) at parieto-occipital areas in the gamma (38–44 Hz) frequency band from −1.5 to −1 s and from −0.75 to 0 s before solution response. Earlier with similar compound remote associate task paradigm, Jung-Beeman and others [39] found (i) higher gamma band activity (center frequency ( *f_c_*) at 39 Hz) at right superior temporal electrode T8 (and surrounding electrodes) from −300 to −20 ms before solution and (ii) increased alpha band activity (*f_c_* = 9.8 Hz) over right parietal-occipital electrode PO8 from −1.31 to −0.56 s. In the present study we also found a strong gamma effect albeit at more posterior regions but no alpha effect. We suggest that there are two primary reasons for these differences. Firstly, we modified the two-alternative forced choice between “Aha!” and no-“Aha!” of Jung-Beeman and others [39] to a 4-point scale rating of suddenness and only compared the solutions with extreme values (i.e. rating of suddenness = 0 versus 3). Thus the results in this study reflect a neural correlate of suddenness, whereas, Jung-Beeman and others [39] investigated the “Aha!” feeling. Secondly, we have used different data pre-processing techniques: raw power values were used in Jung-Beeman and others [39] whereas baseline normalized values were used in this study.

We suggest that the observed gamma effect in the right-hemisphere just before the response reflects retrieval processes when the problem solver successfully retrieves a new solution word. Gamma (and also theta) frequency band activation in right parieto-occipital regions has earlier been associated with retrieval processes from long-term declarative memory [85] and gamma oscillations in general have been related to memory match and utilization processes [86].

### Relationship between Restructuring of the Problem and Suddenness of the Solution

Another interesting issue is the relationship between the restructuring and suddenness of the solution. Behaviorally the problem solvers reported for more than half (55.6%) of the solutions without hint a simultaneous high feeling of suddenness and low involvement of restructuring (chance level lies at 25%, Fig. 1*A*). Actually, the subjective ratings of restructuring and suddenness are negatively correlated (ρ = −0.39). On the one hand, sudden emergence of the solution without any predictable forewarning leading to an “Aha!” is often considered the signature of an insight. On the other hand restructuring the problem in a new way by retrieving relevant but previously unattended information from memory which would eventually lead to a successful solution is considered a necessary component of insight. So how can these two key features of insight be negatively correlated? We have two hypotheses for this intriguing finding on post-hoc basis. The first states that the problem solver would classify a solution to a problem as sudden if the solution was immediately obvious after problem presentation; so restructuring would not be needed and short reaction times would bias the subjective ratings towards high feelings of suddenness. This account was supported by the large and positive linear effects of restructuring rating and negative linear effects of suddenness rating on reaction time. We observed indeed high ratings of suddenness and low involvement of restructuring for trials with shortest reaction times (Fig. 1*C*). Alternatively, when a problem solver experienced a strong feeling of suddenness, the underlying metacognitive processes may be weak and likewise the capability to report about the prior restructuring since the solver was not consciously aware of the ongoing events. Metacognition is an active monitoring process of one's own knowledge and thoughts which is involved in problem solving such as assessing the difficulty of the problem, planning of strategies to approach solution, reviewing or evaluating one's progress, realizing an impasse, recognizing and building new mental representations of the problem or reconsidering one's own thoughts. The metacognitive process got even stronger after hint presentation, since the problem solver was informed on a meta-level that a more appropriate representation was needed [15]. The problem solvers were required to quickly grasp and process the hint to build a new mental representation and would eventually reconsider their own strategies. Actually, the present behavioral data did support this hypothesis. After hint presentation we observed for 84.3% of all correct solutions a high restructuring rating (≥2; Fig. 1*B*), indicating a strong conscious awareness of the ongoing restructuring processes. This explanation also predicts that the frequency of sudden solutions with hint should decrease because of the increased metacognitive processes that are involved after hint presentation. Behavioral results also supported this prediction as the ratings of restructuring and suddenness were uncorrelated for correct post-hint solutions and the rating of suddenness was uniformly distributed between the four possible ratings.

In summary, both of the hypotheses on the underlying mechanisms of restructuring may coexist and are not contradictory. Therefore, we can predict that solutions which evoke a strong feeling of suddenness involve minimal metacognitive processes and unconscious restructuring or better an automatic, subconscious recombination of information which stands in contrast to conscious restructuring which is an attention-demanding process involving executive control.

## Materials and Methods

In this study we characterized data by the mean and SD, if data was normally distributed, and reported median and range otherwise. We assessed the normality of the data by plotting the empirical quantiles of the variable against theoretical quantiles of a Gaussian distribution. The data did not resemble a Gaussian distribution, if the data lay outside of the confidence envelopes based on the standard errors of order statistics for an independent sample from the Gaussian distribution.

### Participants

Twenty one (11 female and 10 male) participants with median age of 26.4 years (range 19.5 to 35.5 years) were paid 25–30 Euro to take part in this experiment. All participants were native English speakers, right-handed, and had normal or corrected to normal vision. The median duration that they had spent in non-English speaking countries was 2 years (range 0 to 21 years) and sixteen of them were monolingual English speakers and five were bilingual. Twelve participants were university students and the others had various professions. All participants were naive to the experiment and research area and gave written informed consent before the start of the experiment. The study was approved by the local ethics committee of the Medical University of Vienna, Austria.

### Experimental paradigm

We used 36 compound remote associate problems of the ones used by Bowden and Jung-Beeman [43], [44] or Jung-Beeman and others [39], modified 96 of them to mainly remove US colloquial terms and developed 6 new problems (see Table S1 for a full list). The initial time limit for each problem was 45 s. The participants were asked to press the left mouse button as soon as they found the solution that they were relatively confident with, however, without mentally checking it. In case the subject experienced within this initial time period a mental impasse or mental block (which we described as “you feel that you cannot proceed” or “you just don't know what else to think of”), she/he pressed the right mouse button as soon as she/he reached this state. Then a clue or a hint appeared on the monitor. Hints disclosed only the first letter (e.g. ’p - - - -’) or uncovered 60 to 75% percent of the letters-always including the first letter (e.g. ’p - - e -’ or ’p a - - r’). The letters that were disclosed were randomized. Following hint presentation the subject had another 6 s to solve the problem. Hints were also provided for trials when the participants could not solve the problem within the allotted time of 45 s [termed as timeout trials].

After receiving solution, we first asked the subject to verbalize the solution and then to provide the following subjective ratings: (1) Rating of Restructuring: Restructuring is a type of event in which one comes to see the problem in a new way. We explained to the subject the following criteria for full restructuring of the problem (rating = 3) [20], [87]: (i) “Elaboration”: one finds a new function of use for a word; (ii) “Re-encoding”: one rejects the use of a word and uses another meaning or application of that word which was not considered previously; (iii) “Constraint Relaxation”: one changes his/her problem representation. The subject rated the extent of restructuring involved in generating the solution on a scale from 0 (no restructuring) to 3 (full restructuring). (2) Rating of Suddenness: The participants were then asked to give a rating of suddenness of the emergence of the solutions on a scale from 0 (the solution appeared gradually as like “slowly dimming up a light bulb”) to 3 (the solution appeared abruptly without any conscious forewarning as like the “flashing of a light bulb”). (3) Rating of Confidence: Finally, we asked how confident the subject was of the solution. The rating of confidence ∈{0, 1, 2, 3} pictures the number of words that the subject is confident with that match with the solution word. In this study trials were only considered when the confidence rating was greater than or equal to 2, thus minimizing the trials that involved significant guessing.

As a control task, compound remote associate problems were presented together with a possible single solution word. The subject had to decide the correctness of the given solution. The time limit for this task was 7 s.

To minimize the required eye movements during each trial (i) participants had to fixate on a fixation cross in the middle of a monitor (Sony Multiscan G520) before initiating a trial by a left mouse button press, and (ii) compound remote associate problems were compactly presented in the middle of the screen with text size 20 and a 24 point line spacing. Trials began with the presentation of three dummy words (‘compound’, ‘compound’, ‘compound’) in place of the actual problem words. This initial pre-trial interval was randomly varied between 1 and 2.5 s. The order of the test words within each problem and the order of the problems within each experiment were randomized, as was the type of problem. Thus the same problem could be a usual compound remote associate task for one subject and a control task for another subject.

Prior to recording, the participants were familiarized with the whole procedure by 5 practice trials. In the actual experiment, the participants solved 100 compound remote associate problems and 33 control trials (of which 22 presented the correct solution word and 11 an incorrect solution). Breaks were provided if necessary and special attention was paid to monitor mental fatigue of the participants. The entire recording was limited to 2 hours for each subject including breaks.

### Behavioral data analysis

Behavioral data was obtained by registering performance and ratings of the constituent features of insightful problem solving on a trial-by-trial basis. Two participants were excluded from further data analysis, because they solved only 50% and 54.5% of all control trials, where the incorrect solution should have been detected and rejected. Thus their data was not trustworthy. Additionally, one particular subject showed a peculiar profile of distribution of response times for mental impasse. This was mainly because this subject pressed the button indicating mental impasse too early (median time 17.3 (9.4–32.9) s) and too often (60% of its trials led to a mental impasse). Thus we excluded two thirds of the shortest mental impasse trials, because short response times and the great mental impasse frequency conjectured unseriousness or laziness of the subject in trying to think of all options to solve the problem. Furthermore, 10% of the shortest mental impasse trials of all participants were excluded-resulting in a cutoff of t = 19.3 s (also for the behavioral data analysis). Statistical testing of possible restructuring and suddenness interaction effects on reaction time, i.e. time to generate solution, was performed by utilizing a linear mixed-effects model [function *lme*() from package nlme in R] which was estimated by Restricted (or Residual) Maximum Likelihood [88], [89], [90], [91]. We implemented the model as a repeated-measure block design with the rating of restructuring and of suddenness as the main within-subject effects and the random effect was due to the subject. We achieved independent and nearly normally distributed residuals by log-transforming the dependent variable, reaction time.

To plot the linear effects (Fig. 1*C*) and thus be able to visually compare the effects between participants, we transformed for each subject the reaction time data, *τ_i_*, *i* = 1, …, *N*, to standardized z-scores, *Z_i_*, *i* = 1, …, *N* where *N* is the number of trials. Therefore we used a robust version of the z-score [92], [93] where the mean and standard deviation (SD) estimates were replaced by the median and the Median Absolute Deviation from the median (*MAD*) with asymptotically normal consistency (i.e. *E*[*MAD*({*τ_i_*}*^N^_i_*_ = 1_)] = *σ* for *τ_i_* distributed as *N(µ*,σ^2^ *)* and large *N*), respectively [function *mad*() in R]:
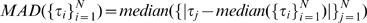


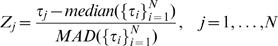



### EEG measurement

Continuous EEG signals were recorded with a Bio-Semi ActiveTwo measurement system (BioSemi, Amsterdam, The Netherlans) by using Ag-AgCl active electrodes. A standard BioSemi head cap which has 32 electrode holders arranged according to the extended International 10–20 System [94] was used. Additionally, horizontal and vertical electrooculogram signals were recorded to monitor blinks and eye movements. All signals were digitized with 24 bit resolution and DC-400 Hz bandwidth and sampled at a sample rate of 2048 Hz.

### EEG data analysis

We performed data preprocessing including artifact correction and time-frequency analysis using complex demodulation [95], [96] with BESA software (MEGIS Software GmbH, Gräfelfing, Germany). EEG signals were average referenced and divided into the following segments: (i) 2.5 s before to 500 ms after the response button press indicating solution, (ii) 3 s time period before the moment of impasse or the point of timeout during the initial 45 s of problem solving and (iii) 200 ms before to 1 s after hint-onset. We chose the last 500 ms of the pre-trial interval as the baseline. Blink artifacts were removed by the two-step surrogate multiple source eye correction method [97], [98] which is a spatial filter approach. In the first step we estimated the prototype of blink topography. We improved the signal-to-noise ratio of the eye artifact pattern search with a 0.5 Hz forward high-pass filter and a zero-phase 8 Hz low-pass filter. Spatial topographies describing the blink artifacts were searched and averaged automatically. The median variance of the first principal component was 99.4% (range 98.2–99.9%). In the second step, brain activity was estimated with a fixed dipole source configuration (4 temporal, 3 parietal, 4 frontal, 3 central and 1 occipital source) while considering the predefined blink artifact topography. We excluded individual trials that still contained eye movements, muscle artifacts or dropped out channels according to the following criteria: (i) maximum amplitudes within the predefined trial segments of interest were higher than 100 µV, (ii) the maximum amplitude difference between two neighboring samples was more than 75 µV or (iii) the maximum signal amplitude was lower than 0.03 µV. Artifact free signals were high pass filtered with 0.2 Hz zero-phase filter (6 dB/octave); power-line noise was eliminated by a 50 Hz notch filter with a 2 Hz bandwidth.

Depending on the correctness of solution, occurrence of mental impasse, utilization of hint and the participants own rating of suddenness and/or restructuring, we categorized and segmented the recorded trials into twelve conditions as follows: (i) correct solution (without hint), (ii) false positive or incorrect solution (without hint), (iii) mental impasse, (iv) timeout (of the initial 45 s), (v) correct solution after mental impasse (post-hint), (vi) timeout after mental impasse (post-hint), (vii) correct solution after initial timeout (post-hint), (viii) post-hint timeout (after initial 45 s timeout), (ix) correct solution with no restructuring (rating of restructuring = 0), (x) correct solution with full restructuring (rating of restructuring = 3), (xi) non-sudden correct solution (rating of suddenness = 0) and (xii) sudden correct solution (rating of suddenness = 3). The twelve categories must not be exclusionary. A problem that is solved correctly without hint, with no restructuring and with a high rating of suddenness would be in categories (i), (ix) and (xii).

For the time-frequency analysis, single trial data corresponding to each condition was transformed into the time-frequency domain using complex demodulation. We chose a time-frequency sampling of 2 Hz/25 ms in the frequency range 4–80 Hz, which corresponded to a full power width at half maximum of 2 · 2.83 Hz and 2 · 39.4 ms. We quantified the event-related changes in spectral power (in %) as follows (Pfurtscheller & Silva, 1999):
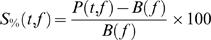
where *P(t, f )* is the arithmetic mean of the spectral power of all trials for a specific time *t* and frequency *f* and *B( f )* is the mean spectral power at frequency *f* over the 500 ms long baseline for all trials. Thus *S_%_(t, f)* is the percentage change of task related power with respect to the baseline power at a specific frequency [99]. These changes have been called event-related synchronization (ERS) for higher task-related power or event-related desynchronization (ERD) for lower task-related power as compared to the baseline; ERS and ERD are due to an increase or a decrease, respectively, in local synchrony of the underlying neuronal populations oscillating in the same frequency band. For each condition, only those participants were included that produced at least two artifact-free trials. We calculated *S_%_(t, f)* for each condition and subject and then transformed ERS values in percentage to a ratio expressed in decibel by
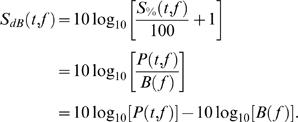
By this instantaneous differencing, the distribution of ERS values was rendered approximately Gaussian, which made the subsequent parametric t-tests on log-transformed differences valid and exact [100]. The subsequent statistical analysis was performed with mean *S_dB_* values for time-windows spanning 200 ms for the time-locked (to hint presentation) comparisons and 500 ms for all other comparisons and two successive windows overlapped by half of the window length. Time-frequency data was also averaged within standard frequency bands as follows: theta (4–8 Hz), alpha1 (8–10 Hz), alpha2 (10–12 Hz), beta1 (12–20 Hz) and beta2 (20–30 Hz). For gamma band, the frequency boundaries were chosen on the basis of visual inspection of the averaged ERS values [85].

To avoid the multiple comparisons problem and to be able to effectively control the type I error rate, we used a nonparametric cluster randomization test [85], [101], [102] for establishing the significance of the time-frequency power difference between any two compared conditions. For each time, frequency and electrode combination, paired two-sided t-test statistics were calculated. We only considered significant values in the subsequent analysis that were smaller than a pre-specified threshold of *p* = 0.01 (two-sided), or *p* = 0.05 if mentioned. Next we searched for both positive and negative t-statistic clusters in time, frequency and electrode space, where we considered electrodes with a distance of less than 7 cm as neighbors (yielding on average 6.8 neighbors per channel). This approach seems justified since neighboring electrodes record from overlapping neural sources. We assumed that a robust cluster should encompass at least 4 neighboring channels. In case the cluster was localized at the edge of the scalp, 3 neighboring channels within one cluster were presumed as sufficient. For each cluster we calculated the sum of the t-test statistics as the test statistic. Subsequently, Monte Carlo randomization was used to obtain a distribution function of the test statistics. Thus, the entire analysis (t-tests, thresholding, finding clusters, computing the test statistic) was repeated for each randomization, and maximum and minimum t-sum values were repetitively saved. The total number of randomizations was 1900, with which we accomplished that the coefficient of variation of the test statistic varied less than 10% [103]. If this number exceeded the maximum possible number of permutations an exact permutation test was conducted (for *N_S_*≤10). The significance *P* of an original cluster was estimated according to the proportion of the randomization null distribution exceeding the observed maximum/minimum t-sum test-statistics.

## Supporting Information

Table S1 List of used compound remote associate problems and their solvability in the study. The data is presented in descending order according to the percentage of subjects solving the compound remote associate problem within the 45-s time limit. Words in bold correspond to compound remote associate problem changes compared to the initially published list by Bowden and Jung-Beeman [43].
(0.27 MB RTF)

Table S2 Percentage of correct and incorrect trials. Mean (SD) percentage of compound remote associate problems for which the subjects gave correct or incorrect (false positive) solutions without and with hint.
(0.02 MB RTF)
