# Supplementary material for: Deconstructing Insight: EEG Correlates of Insightful Problem Solving
Source: PLoS One. 2008 Jan 23;3(1):e1459. doi: 10.1371/journal.pone.0001459 (PMC2180197; doi:10.1371/journal.pone.0001459)
Supplement: Table S1 — List of used compound remote associate problems and their solvability in the study. The data is presented in descending order according to the percentage of subjects solving the compound remote associate problem within the 45-s time limit. Words in bold correspond to compound remote associate problem changes compared to the initially published list by Bowden and Jung-Beeman [43]. (0.27 MB RTF) [file pone.0001459.s001.rtf]

Table S1
List of used compound remote associate problems and their solvability in the study. 
	
Compound Remote Associate Problem	Solution	% of Subj. Solving Item (pre-hint)	Mean Solution Time (ms)	SD	N	
food/forward/break	fast	100%	6085	3504	10	
worm/shelf/mark	book	94%	5272	2458	17	
flag/north/position	pole	92%	4777	2936	12	
palm/line/house	tree	86%	7430	5151	14	
night/wrist/stop	watch	85%	5898	3741	13	
wheel/hand/shopping	cart	83%	4954	1354	12	
high/mate/teacher	school/(class)	83%	8670	4197	12	
light/birthday/stick	candle	80%	12003	8711	10	
sandwich/house/golf	club	80%	13677	7419	10	
hard/drift/chopper	wood	79%	10696	8452	14	
river/note/account	bank	78%	11378	9386	9	
fur/hanger/tail	coat	76%	8228	5011	17	
pain/serial/whale	killer	75%	10424	7901	16	
cane/daddy/free	sugar	73%	8791	6085	11	
dust/cereal/toilet	bowl	71%	7288	8268	14	
pine/pie/juice	apple	70%	8844	4811	10	
health/taker/child	care	70%	7382	7431	10	
tooth/stroke/hair	brush	69%	5940	5551	16	
safety/cushion/point	pin	69%	5382	2629	16	
equal/stop/language	sign	69%	13316	8121	13	
fire/ranger/tropical	forest	67%	8428	7147	15	
chamber/mask/natural	gas	67%	6323	3051	15	
carpet/alert/cross	red	67%	10216	6646	15	
measure/worm/video	tape	67%	9382	4799	15	
boat/limit/high	speed	67%	7506	6703	9	
sleeping/pipe/trash	bag	65%	9383	7044	17	
rocking/wheel/man	chair	65%	9136	4765	17	
wise/work/alarm	clock	65%	8468	4626	17	
dream/break/light	day	65%	5097	3770	17	
cottage/knife/cake	cheese	64%	3662	2075	11	
house/grass/card	green	64%	8853	4310	14	
dew/moon/bee	honey	62%	11280	8934	16	
right/cat/carbon	copy	60%	4952	1692	10	
back/clip/wall	paper	60%	10927	8957	10	
pad/trap/click	mouse	60%	8051	8536	15	
gun/puff/room	powder	58%	10130	4706	12	
flake/mobile/fall	snow	57%	10752	7846	14	
date/alley/fold	blind	56%	8605	6180	16	
water/mine/shaker	salt	56%	6782	4309	16	
polish/finger/head	nail	56%	8116	4479	9	
cry/walker/face	baby	56%	8564	5339	16	
end/line/brain	dead	55%	7090	3738	11	
wet/law/case	suit	54%	7940	5067	13	
party/towel/ball	beach	54%	8204	4307	13	
fish/mine/rush	gold	53%	7161	6864	19	
bed/throat/point	sore	53%	15310	9648	15	
fog/car/shoe	horn	50%	11526	11136	12	
path/print/bare	foot	47%	9857	5429	15	
egg/collar/wash	white	47%	4197	1221	15	
stick/maker/point	match	46%	4775	2164	13	
land/dairy/house	farm	44%	14676	7102	16	
black/man/peep	hole	44%	13582	7468	9	
time/capsule/ship	space	44%	12293	8365	9	
through/disk/way	drive	44%	9752	4360	9	
age/mile/sand	stone	43%	7360	3350	14	
main/sweeper/light	street	43%	7740	2120	7	
artist/fire/route	escape	42%	7628	2706	12	
stand/child/piano	grand	42%	14817	10675	19	
tank/hill/secret	top	41%	9635	6162	17	
cross/list/mate	check	40%	6170	1711	15	
catcher/fight/hot	dog	40%	16563	7895	15	
foster/name/album	family	40%	12929	6774	15	
father/head/stick	figure	40%	5017	1067	10	
skirts/black/put	out	39%	14404	8709	18	
air/board/dollar	bill	38%	8087	2333	8	
self/attorney/minister	defenc(/s)e	38%	11111	8382	8	
teeth/alarm/start	false	38%	9165	5774	13	
way/water/flood	gate	38%	16429	6485	16	
petroleum/bean/fish	jelly	38%	5750	2677	13	
officer/cash/crime	petty	38%	9206	8138	13	
man/glue/star	super	38%	11543	5442	8	
cat/number/phone	call	36%	13301	6892	11	
maker/order/pocket	money	36%	13036	11527	14	
bath/dial/flower	sun	36%	11261	9388	11	
shock/shave/noon	after	33%	8772	9225	15	
cross/rain/tie	bow	33%	8157	5142	6	
flower/luck/belly	pot	33%	9827	3046	9	
term/circuit/hand	short	33%	9465	9133	15	
illness/bus/computer	terminal	33%	15951	6360	12	
letter/building/buster	block	33%	7141	4742	6	
wave/shield/stroke	heat	33%	9171	6213	15	
play/cow/friend	boy	33%	10541	7946	12	
quick/spoon/screen	silver	31%	9220	6777	16	
note/chain/master	key	30%	7616	2339	10	
over/market/room	stock	29%	7800	5247	14	
type/ghost/screen	writer	27%	8920	8057	15	
tomato/board/tooth	paste	27%	21369	4844	15	
ice/cream/war	cold	25%	6651	3821	16	
mill/tooth/dust	saw	25%	5754	5202	16	
man/cat/sleep	walk	25%	17921	5917	16	
pea/shell/chest	nut	24%	14050	9896	17	
home/sea/bed	sick	23%	8431	6018	13	
sense/wealth/place	common	21%	14535	11364	14	
flower/friend/scout	girl	21%	10680	5925	14	
board/toggle/back	switch	21%	10501	7027	14	
line/patrol/town	border	21%	6707	3222	14	
home/boat/oil	motor	21%	11176	9205	14	
child/true/letter	love	20%	17789	10789	10	
horse/human/course	race	18%	12125	4733	17	
ear/tone/finger	ring	18%	4655	1165	11	
spoon/cloth/time	table	18%	9502	7632	11	
cover/arm/wear	under	18%	10177	10904	17	
play/ground/weather	fair	17%	6328	3901	12	
head/shade/post	lamp	17%	8184	3609	12	
tomato/blossom/sour	cherry	15%	7203	4244	13	
hard/bottom/garden	rock	14%	8209	293	14	
property/moral/judgment	value	13%	11459	6608	15	
cry/field/ship	battle	12%	2685	-	8	
fire/summer/ground	camp	12%	17856	16729	17	
bump/egg/step	goose	12%	9544	820	16	
reading/service/stick	lip	12%	17885	4284	17	
iron/boat/engine	steam	12%	3249	-	8	
control/place/rate	birth	10%	5237	-	10	
shadow/contact/ball	eye	10%	9619	-	10	
tooth/potato/heart	sweet	10%	15739	-	10	
life/body/post	guard	10%	4709	-	10	
home/arm/room	rest	8%	4195	-	12	
jury/display/side	panel	8%	16257	-	13	
line/blue/rocket	sky	7%	6595	-	14	
forward/line/away	straight	7%	13839	-	14	
animal/back/mud	pack	6%	17603	-	18	
rain/test/ant	acid	0%	-	-	17	
cast/side/band	broad	0%	-	-	14	
lounge/hour/party	cocktail	0%	-	-	10	
break/bean/black	coffee	0%	-	-	10	
wise/king/meat	crab	0%	-	-	17	
keeper/mind/show	game	0%	-	-	14	
knife/light/ball	pen	0%	-	-	14	
fall/arm/coal	pit	0%	-	-	16	
point/station/horse	power	0%	-	-	7	
line/fruit/card	punch	0%	-	-	13	
oil/bar/tuna	salad	0%	-	-	14	
opera/hand/box	soap	0%	-	-	13	
computer/cable/access	network	0%	-	-	15	
perfect/life/march	past	0%	-	-	13	
pistol/car/shopping	toy	0%	-	-	15	
read/water/child	proof	0%	-	-	16	
away/long/down	run	0%	-	-	7	
						
The data is presented in descending order according to the percentage of subjects solving the compound remote associate problem within the 45-s time limit. Words in bold correspond to compound remote associate problem changes compared to the initially published list by Bowden and Jung-Beeman [43].	
