# Supplementary material for: Deconstructing Insight: EEG Correlates of Insightful Problem Solving
Source: PLoS One. 2008 Jan 23;3(1):e1459. doi: 10.1371/journal.pone.0001459 (PMC2180197; doi:10.1371/journal.pone.0001459)
Supplement: Table S2 — Percentage of correct and incorrect trials. Mean (SD) percentage of compound remote associate problems for which the subjects gave correct or incorrect (false positive) solutions without and with hint. (0.02 MB RTF) [file pone.0001459.s002.rtf]

Table S2
Mean reaction times for specific ratings of suddenness and restructuring for correct solutions without hint.	
RoS	0	1	2	3	
RoR					
0	9.7 (7.7)	11.1 (8.5)	6.8 (2.6)	4.0 (1.5)	
1	11.7 (4.5)	12.4 (6.2)	10.3 (3.7)	7.1 (2.7)	
2	27.5 (10.4)	19.2 (7.9)	12.5 (5.1)	8.5 (6.1)	
3	25.7 (12.7)	22.8 (7.2)	15.4 (9.0)	20.8 (13.8)	
					
Mean (SD) reaction times in seconds for 19 subjects. The longer the required solution time for a correct solution was (i) the higher was the subjective rating of restructuring and (ii) the lower was the suddenness feeling.	
